# Parthenolide promotes the repair of spinal cord injury by modulating M1/M2 polarization via the NF-κB and STAT 1/3 signaling pathway

**DOI:** 10.1038/s41420-020-00333-8

**Published:** 2020-10-06

**Authors:** Tao Gaojian, Qian Dingfei, Li Linwei, Wang Xiaowei, Zhou Zheng, Liu Wei, Zhu Tong, Ning Benxiang, Qian Yanning, Zhou Wei, Chen Jian

**Affiliations:** 1 Department of Anesthesiology, The First Affiliated Hospital of Nanjing Medical University, Nanjing, 210029 China; 2 Department of Pain Management, Affiliated Drum Tower Hospital, Medical School of Nanjing University, Nanjing, 210008 China; 3 Department of Orthopedic, The First Affiliated Hospital of Nanjing Medical University, Nanjing, 210029 China; 4 Department of Orthopedic, The Third Affiliated Hospital of Soochow University, Changzhou, 213003 China

**Keywords:** Microglia, Spinal cord diseases

## Abstract

Spinal cord injury (SCI) is a severe neurological disease; however, there is no effective treatment for spinal cord injury. Neuroinflammation involves the activation of resident microglia and the infiltration of macrophages is the major pathogenesis of SCI secondary injury and considered to be the therapeutic target of SCI. Parthenolide (PN) has been reported to exert anti-inflammatory effects in fever, migraines, arthritis, and superficial inflammation; however, the role of PN in SCI therapeutics has not been clarified. In this study, we showed that PN could improve the functional recovery of spinal cord in mice as revealed by increased BMS scores and decreased cavity of spinal cord injury in vivo. Immunofluorescence staining experiments confirmed that PN could promote axonal regeneration, increase myelin reconstitution, reduce chondroitin sulfate formation, inhibit scar hyperplasia, suppress the activation of A1 neurotoxic reactive astrocytes and facilitate shift from M1 to M2 polarization of microglia/macrophages. To verify how PN exerts its effects on microglia/macrophages polarization, we performed the mechanism study in vitro in microglia cell line BV-2. PN could significantly reduce M1 polarization in BV2 cells and partially rescue the decrease in the expression of M2 phenotype markers of microglia/macrophage induced by LPS, but no significant effect on M2 polarization stimulated with IL-4 was observed. Further study demonstrated PN inhibited NF-κB signal pathway directly or indirectly, and suppressed activation of signal transducer and activator of transcription 1 or 3 (STAT1/3) via reducing the expression of HDAC1 and subsequently increasing the levels of STAT1/3 acetylation. Overall, our study illustrated that PN may be a promising strategy for traumatic SCI.

## Introduction

Spinal cord injury (SCI) is defined as damage to the spinal cord that temporarily or permanently causes the functional loss including movement or sensation, resulting in decreased quality of life, persistently increased lifelong mortality rates, and significant medical burdens to family and society^1,2^. In SCI, primary injury mainly damaged the spinal cord directly, leading to disrupting the vasculature and compromising the blood–spinal cord barrier. Together, these events immediately initiate a complex secondary injury cascade with several complex phenomena, such as hemorrhage and ischemia, hypoxia, inflammation and edema^2^. Among all the mechanisms of secondary damage, inflammation is the most important, and directly or indirectly controls the sequelae after SCI^3^. The majority of cellular elements in central nervous system (CNS) are constituted by neurons and glia (astrocytes, ependymal, oligodendrocytes and microglia) cells. After SCI, neuroinflammation is primarily mediated by microglia cells, which are the main components of the innate immune system and regarded as resident macrophages in the CNS^4–6^. Due to the destruction of blood–spinal cord barrier (BBB) and blood vessel injury, peripheral macrophages, neutrophils and lymphocytes are recruited in damaged tissues under the influence of local inflammatory factors, chemokines, and vasoactive peptides with the synergy of firstly microglia activation^2,4^. Because microglia and peripheral macrophages have myelomonocytic origin, therefore peripheral macrophages adopt many of the markers and behaviors of microglia^7,8^. These similarities make it difficult to distinguish between them in spinal cord injury foci.

Under the stimulus of the lesion microenvironment of spinal cord, microglia/macrophages polarize into two phenotypes: a classically activated pro-inflammatory (M1) phenotype and an alternatively activated anti-inflammatory (M2) phenotype^9^. The ratio of M1 to M2 maintains the homeostasis of the local microenvironment. M1 phenotype, is associated with the production of proinflammatory cytokines such as tumor necrosis factor-α (TNF-α), interleukin-1 beta (IL-1β), interleukin-6 (IL-6), glutamate, superoxide, nitric oxide (NO), reactive oxygen species (ROS), chemokines, and proteases^3,4,10^. These inflammatory mediators initiate cascades of neurotoxic responses in secondary phase of SCI and can cooperate to contribute to the preponderance of damage (apoptosis and necrosis) to endothelia, neurons, axons and oligodendrocytes, and finally phagocytosis. In contrast, M2 phenotype is symbolized by the release of anti-inflammatory cytokines (IL-4, IL-10, IL-13), transforming growth factor beta (TGF-β), and several neurotrophic factors, such as ciliary neurotrophic factor (CNTF), insulin-like growth factor (IGF), epidermal growth factor (EGF), and nerve growth factor (NGF), all of which antagonize the pro-inflammatory responses and facilitate neuroregeneration, particularly axonal extension, after CNS injury^3,4,10^. Therefore, Inhibiting M1 or promoting M2 polarization may be a promising treatment strategy to facilitate functional recovery after SCI. Additionally, M1 phenotype of microglia/macrophages are quickly activated in the first few days and continue to be activated 28 days after injury; however, within 7 days, transient activation of a small number of M2 macrophages/microglia are observed^11,12^. This is a critical time for spinal cord injury repair with one month of the injury, and to be clinically effective, targeting M1/M2 polarization balance needs depend on the time window.

Parthenolide (PN), as a sesquiterpene lactone used for therapy of inflammation, via inhibiting canonical NF-κB (p65/p50) activation followed by direct interaction of PN with inhibitor of nuclear factor kappa-B kinase (IKK) β and subsequently suppressing IKK signalsome^13,14^, as well as an inductor of HDAC1 degradation by proteasome activity^15^ has been reported to have anti-cancer property^16,17^. Furthermore, PN has also been considered to be involved in the regulation of signal transducer and activator of transcriptions (STATs) signaling pathway^18,19^, but the mechanism is still unclear. Both the NF-κB and STATs signals are considered as important pathways in regulating microglia/macrophages polarization. However, the ability of PN to regulate M1/M2 polarization and its neuroprotective effect in SCI remain unknown. This study aimed to investigate whether PN could regulate M1/M2 polarization and promote functional recovery of mice after SCI as well as the underlying mechanisms.

Inspiringly, we found that PN could improve functional recovery in SCI mice by promoting axonal regeneration, increasing myelin reconstitution, reducing chondroitin sulfate proteoglycan (CSPG) formation, inhibiting scar hyperplasia, and suppressing the activation of A1 neurotoxic reactive astrocytes. In addition, we also showed that M2 polarization of microglia/macrophage was activated and M1 polarization was suppressed by PN in vivo. In vitro, PN mainly inhibited the M1 polarization of BV2 cells induced by LPS accompanied by a reversal of M2 phenotype and had no effect on M2 polarization induced by IL-4. Further study demonstrated that PN regulated M1/M2 polarization balance mainly via inhibiting NF-κB signal pathway directly or indirectly, and suppressing activation of STAT1/3 via the reduced expression of HDAC1 and the subsequently increasing levels of STAT1/3 acetylation. This study demonstrates that PN may be a promising therapeutic drug to target microglia polarization in SCI.

## Results

### PN treatment improved functional recovery following traumatic SCI

**Fig. 1:**
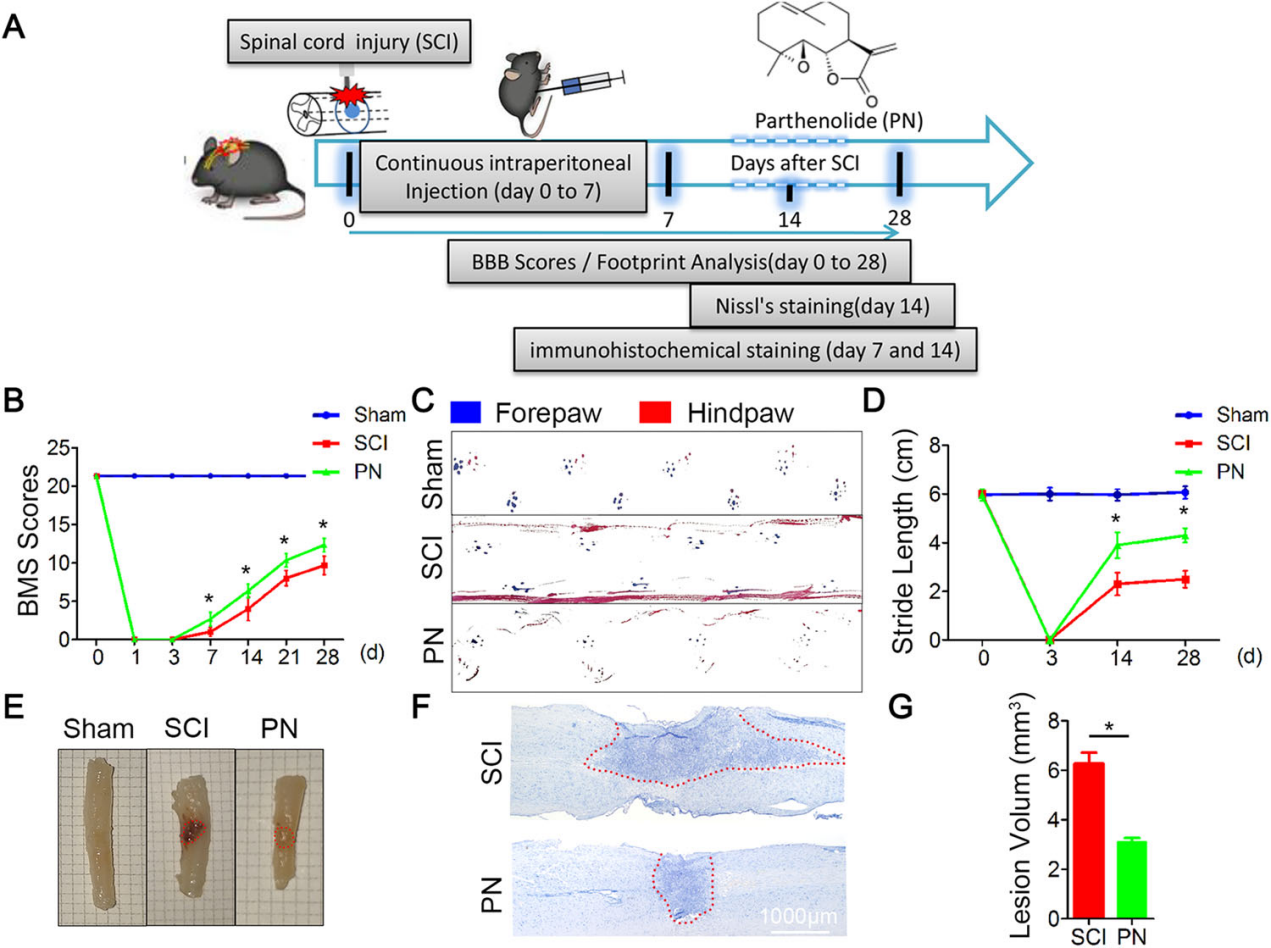
PN promoted neurological function recovery after SCI. **a** Schematic diagram of experimental design. PN were intraperitoneally injected within 10 min after weight drop at the T10 level, and then continuously injected daily until 7 days after injury. **b** Basso, Beattie, and Bresnahan (BBB) limb function scores at different times after spinal cord contusion. **c** Representative footprints of an animal walking 14 days after SCI. Blue: forepaw print; red: hindpaw print. **d** Quantitative analysis of the footprint (**c**) in the two groups. **e** Gross morphology of spinal sections on day 14 postinjury. The boundary of the traumatic lesion area is indicated by the dashed lines. **f** Representative Nissl-stained sagittal section of spinal cord on day 14 postinjury. Scale bar, 1000 μm. **g** Quantitative analysis of lesion volume (**f**) in the two groups. All data are presented as means ± SEM (*n* = 5 mice per group). *P < 0.05.

To test the neuroprotective efficacy of PN in vivo, we performed motor function assessments as well as histological injury study in mice with weight-induced spinal contusion at T10 (Fig. 1a). Within 10 min posttrauma, we intraperitoneally administered PN and then continuously injected daily until 7 days considering the blood–brain barrier (BBB) closing time after injury^20^. Motor function in the SCI group and the PN group gradually improved over the first week following SCI as evidenced by BMS scores. Mice treated with PN showed significantly better performance than the controls beginning at day 7 and maintained this superiority over the remainder of the study (Fig. 1b). Coordination of forepaw–hindpaw movements decreased significantly immediately after SCI as determined by gait analysis, but animals treated with PN showed significantly faster gait recovery and improved motor coordination compared with SCI group animals (Fig. 1c, d). As shown by the gross morphology of the injured spinal cords, the traumatic lesion area (brown colored region) on the spinal cord was visible (Fig. 1e). After treatment with PN, the lesion area was notably smaller than that of the SCI only group. Additionally, it should be noted that motor performance and the gross morphology of the sham-surgery group remained unchanged throughout the test period. Nissl staining revealed a significant loss of SCI tissue in the SCI group at 2 weeks post injury that was significantly reduced in the PN treatment group (Fig. 1f, g). Collectively, these data suggest that PN promotes the functional motor recovery following SCI.

### PN treatment inhibited demyelination and promoted axon regeneration following SCI

**Fig. 2:**
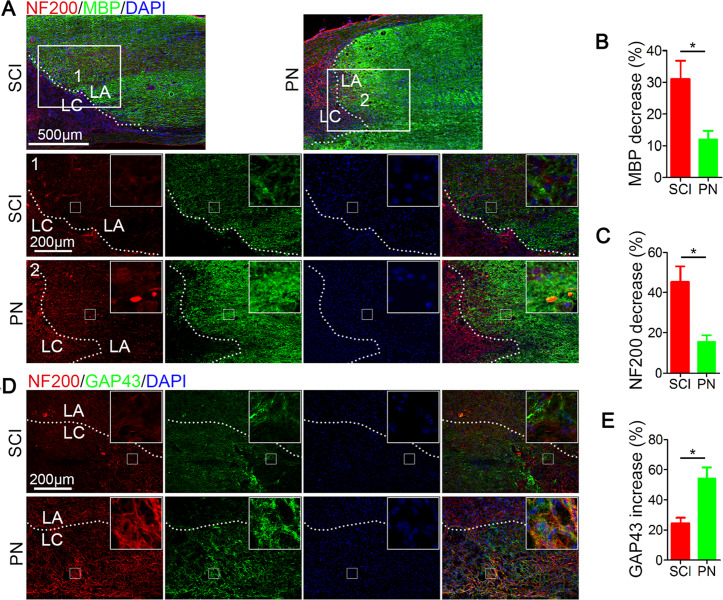
PN suppressed demyelination and promoted axon regeneration after SCI. **a** Representative images of NF200 (red) and MBP (green) immunohistochemical staining on day 14 after injury in spinal cord lesion areas. Two columns (1, and 2 respectively represent SCI group and PN group) are the enlarged images of the area around the damage boundary. Lesion core (LC) and lesion adjacent (LA) of the cavity is distinguished by the dashed lines. All cell nuclei were counterstained with DAPI (blue). Scale bar, 500 μm (upper) and 200 μm (below). **b**, **c** Semiquantification of MBP and NF200 intensity decrease in **a**. **d** Representative images of NF200 (red) and GAP43 (green) immunohistochemical staining on day 14 after injury in the two groups. All cell nuclei were counterstained with DAPI (blue). Scale bar, 200 μm. **e** Semiquantification of GAP43 intensity increase in **d**. All data are presented as means ± SEM (*n* = 5 mice per group). *P < 0.05.

To assess whether PN could suppress demyelination, sections from mouse spinal cords were examined on 14 day postinjury for myelin basic protein (MBP)^21,22^. The decrease in the staining against MBP at the edge of the lesion areas compared with the distant area, as assessed by average pixel intensity values, was much lower in the PN group than in the SCI group (Fig. 2a, b). What’s more, new myelin sheaths even appeared in the lesion center areas. Neuroflaments are cell type specific proteins in central nerve system and qualified as potential surrogate markers of damage to neuron and axon^23^. The immunostaining analysis of 200 kDa subunit of neuroflament (NF200), which contributes to anomalous electrophoretic mobility, has been widely employed to evaluate the neuron and axon damage^24,25^. In comparison with the SCI only group, a significant increase in NF200 labeling adjacent to the injury site was observed for PN treated group, suggesting the continuous neuronal protection effect of PN (Fig. 2a, c). With respect to axonal regrowth^26^, immunostaining using an antibody against GAP43, a marker for regenerating axons, showed that the expression of GAP43 was significantly increased both at the lesion epicenter in mice treated with PN in comparison with SCI group (Fig. 2d, e). Besides, NF200-positive fibers in mice treated with PN were more likely to be reproduced at the injury center, and most of these fibers expressed GAP43 according to the merge image between NF200 staining with GAP43 staining (Fig. 2d). These results indicate that PN reduces the demyelination, and promote axonal regeneration in the injured lesion area.

### PN suppressed glial scar formation, CSPG production, and the activation of A1 neurotoxic reactive astrocytes after SCI

**Fig. 3:**
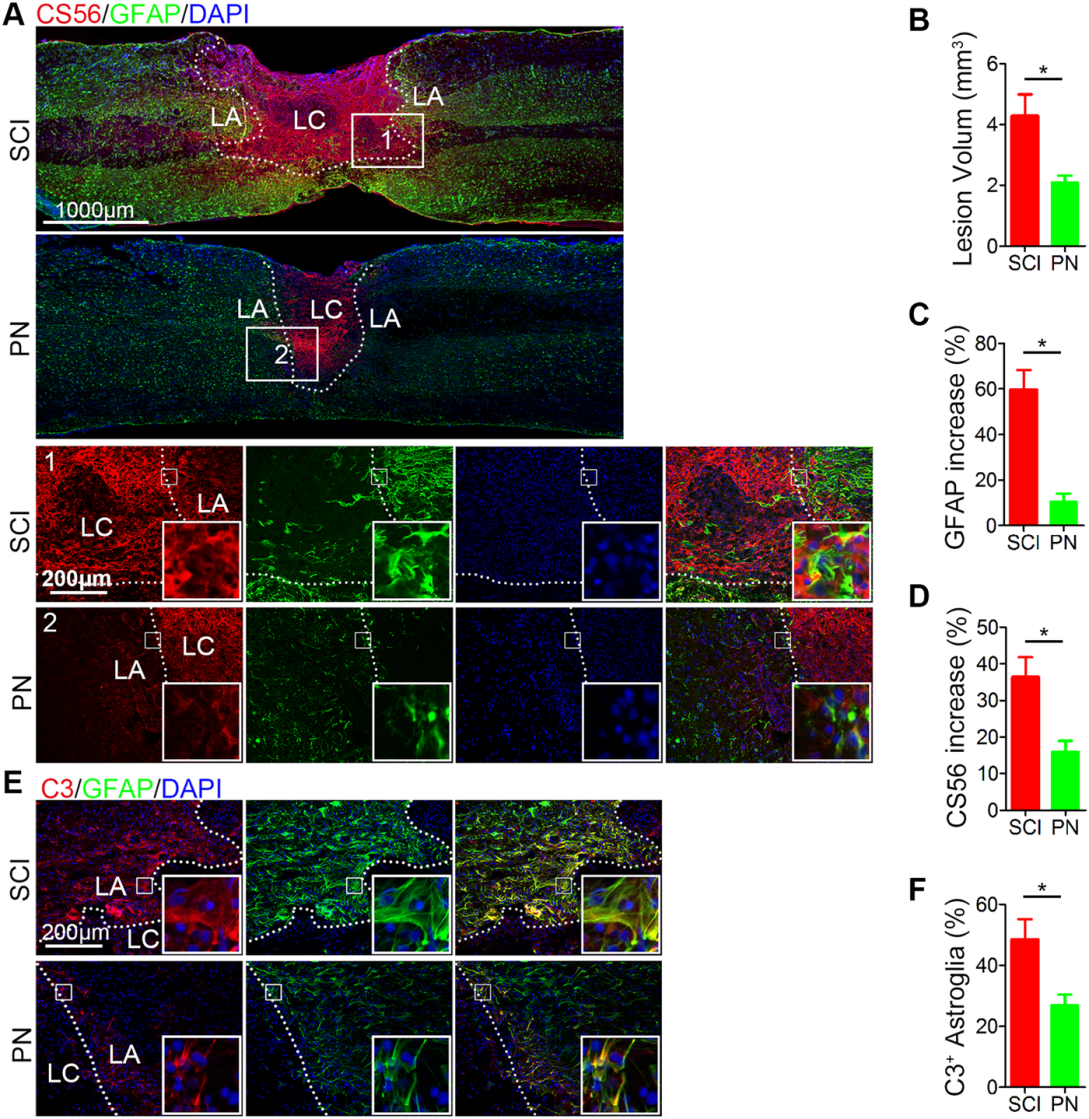
PN inhibited the formation of astrocyte scar, reduced the production of CSPG and attenuated the activation of A1 astrocytes after SCI. **a** Representative images of CSPG detected by CS56 (red) and GFAP (green) immunohistochemical staining on day 14 after injury in spinal cord lesion areas. Two columns (1, and 2 respectively represent SCI group and PN group) are the enlarged images of the area around the damage boundary. The cavity boundary is indicated by the dashed lines. All cell nuclei were counterstained with DAPI (blue). Scale bar, 1000 μm (upper) and 200 μm (below). **b** Quantitative analysis of lesion volume according to GFAP-positive scar (**a**) in the two groups. **c**, **d** Semiquantification of CS56 and GFAP intensity increase in (**a**). **e** Representative images of C3 (red) and GFAP (green) immunohistochemical staining on day 14 after injury in the two groups. All cell nuclei were counterstained with DAPI (blue). Scale bar, 200 μm. **f** Quantitative analysis of the ratio of C3^+^/GFAP^+^ cells in the lesion in (**e**). LA, lesion core; LA, lesion adjacent. All data are presented as means ± SEM (*n* = 5 mice per group). *P < 0.05.

Many studies showed that glial scar which is mainly composed of reactive astrocytes with the cellular hypertrophy and the increasing expression of glial fibrillary acidic protein (GFAP) is not only a mechanical barrier to impede axon regeneration^27–30^, but also secretes CSPGs regarded as the principal inhibitors of axon regeneration to form a chemical barrier^31–33^. Firstly, we found that the cavity volume after SCI via calculating the area bounded by the glial scar staining with GFAP, was significantly reduced after PN treatment (Fig. 3a, b). Regarding the SCI group, the region in proximity to the lesion area was characterized by hypertrophic astrocytes with multiple GFAP^+^ processes for day 14 postinjury; however, in the PN group, peritraumatic astrocytes were morphologically indistinguishable from astrocytes located distal to the injury site at the same time postinjury (Fig. 3a). The GFAP immunoreactivity near the injury site in spinal cord of PN-treatment mice was lower than that of only-SCI mice (Fig. 3a, c). Total CSPG levels determined by dot blot with CS56 antibody^33,34^ were significantly higher in SCI lesions, and were significantly reduced by disruption of astrocytic scar formation after the treatment with PN (Fig. 3a, d). As previously reported, astrocytes could be divided into two different types, named A1 astrocytes and A2 astrocytes^35^. In particular, complement component 3 (C3) overexpression is a characteristic feature of A1 astrocytes^35^ and we identified A1 astrocytes by co-immunofluorescence staining for C3 and GFAP. Immunostaining of spinal cord tissue revealed a significant decrease in A1 astrocyte number at the lesion area after PN administration on day14 postinjury compared to that in injured spinal cord from the control group (Fig. 3e, f).

### PN treatment reduced inflammatory infiltration, and induced the transformation of microglia/macrophages from M1 phenotype to M2 phenotype in lesion area after SCI

**Fig. 4:**
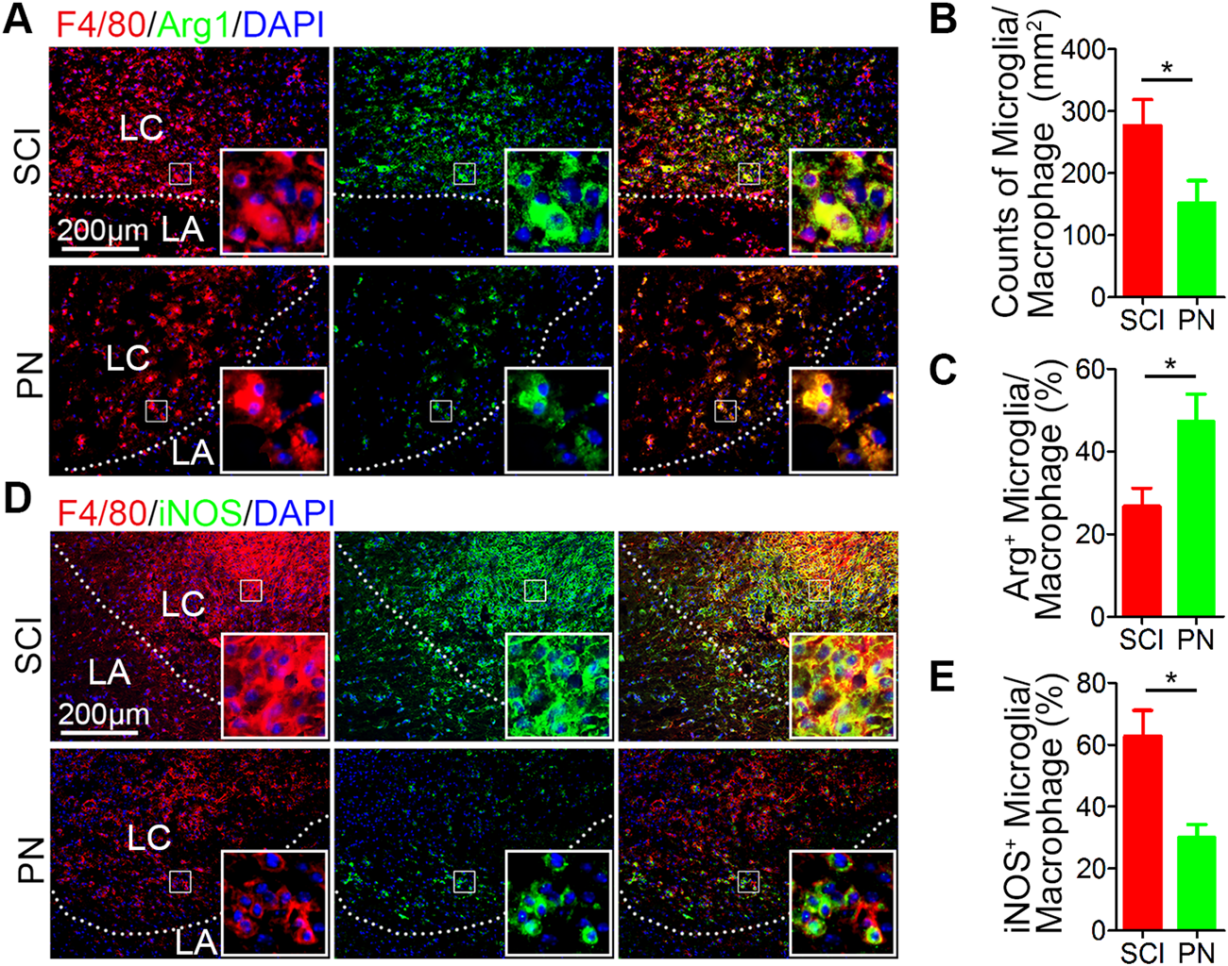
PN inhibited microglia infiltration and shifted M1/M2 polarization after SCI. **a** Representative images of F4/80 (red) and Arg1 (green) immunohistochemical staining on day 7 after injury in spinal cord lesion areas. The cavity boundary is indicated by the dashed lines. All cell nuclei were counterstained with DAPI (blue). Scale bar, 200 μm. **b**, **c** Quantitative analysis of the number of F4/80^+^ microglia/macrophages and the ratio of Arg1^+^/F4/80^+^ microglia/macrophages in the lesion in (**a**). **d** Representative images of F4/80 (red) and iNOS (green) immunohistochemical staining on day 7 after injury in spinal cord lesion areas. The cavity boundary is indicated by the dashed lines. All cell nuclei were counterstained with DAPI (blue). Scale bar, 200 μm. **e** Quantitative analysis of the ratio of iNOS^+^/F4/80^+^ microglia/macrophages in the lesion in **d**. LA, lesion core; LA, lesion adjacent. All data are presented as means ± SEM (*n* = 5 mice per group). *P < 0.05.

Following SCI, microglia/macrophages on sensing cues of cellular or tissue damage in their niche, become activated, undergo hypertrophic morphological and functional changes, as well as transform to a migratory mode^4^. On the one hand, PN has anti-inflammatory effects;^13,16,17^ on the other hand, our previous results (Fig. 3e, f) showed that PN reduced A1 neurotoxic reactive astrocytes, which could be induced by activated microglia^35^. Therefore, we observed the infiltration of microglia/macrophage in spinal cord injury tissues by labeling with F4/80. It showed that the microglia/macrophages in the lesion area were decreased after PN treatment (Fig. 4a, b). Traditionally, microglia/macrophages (save for the quiescent and state-ramified morphology) exist in two basic polarized states, which are dependent on external signals, the M1 (classical activated) pro-inflammatory phenotype and M2 (alternatively activated) anti-inflammatory phenotype, maintaining spinal cord homeostasis^36^. Activated M1 microglia/macrophages, result in severe destruction of spinal cord tissue cells, such as endothelia, neurons, axons and oligodendrocytes^3,4,6^, whereas M2 microglia/macrophages play an important role in anti-inflammatory function, neuro/axonal-trophic support and scar-degrading capacities^3,4,10^. Therefore, in order to further explore the specific mechanism of PN promoting neurological function recovery, we evaluated the characteristic polarization of microglia/macrophages after SCI in different groups using the representative M1-assocaited iNOS and M2-associated Arg1 markers for double immunofluorescent staining together with F4/80, which detects microglia/macrophage in the spinal cord. As shown in Fig. 4a, c–e, there was a marked decrease in the iNOS-positive microglia/macrophage and a higher level of Arg1 in the microglia/macrophages was observed in the lesion areas on day 7 postinjury in the PN group compared with the SCI group. Consequently, these results demonstrate that PN has a significant effect on inflammatory infiltration and the ratio of anti-inflammatory to pro-inflammatory phenotype after SCI via shifting microglial/macrophages polarization from M1 to M2 phenotype.

### PN shifted the microglia from M1 to M2 phenotype in BV2 cells in vitro

**Fig. 5:**
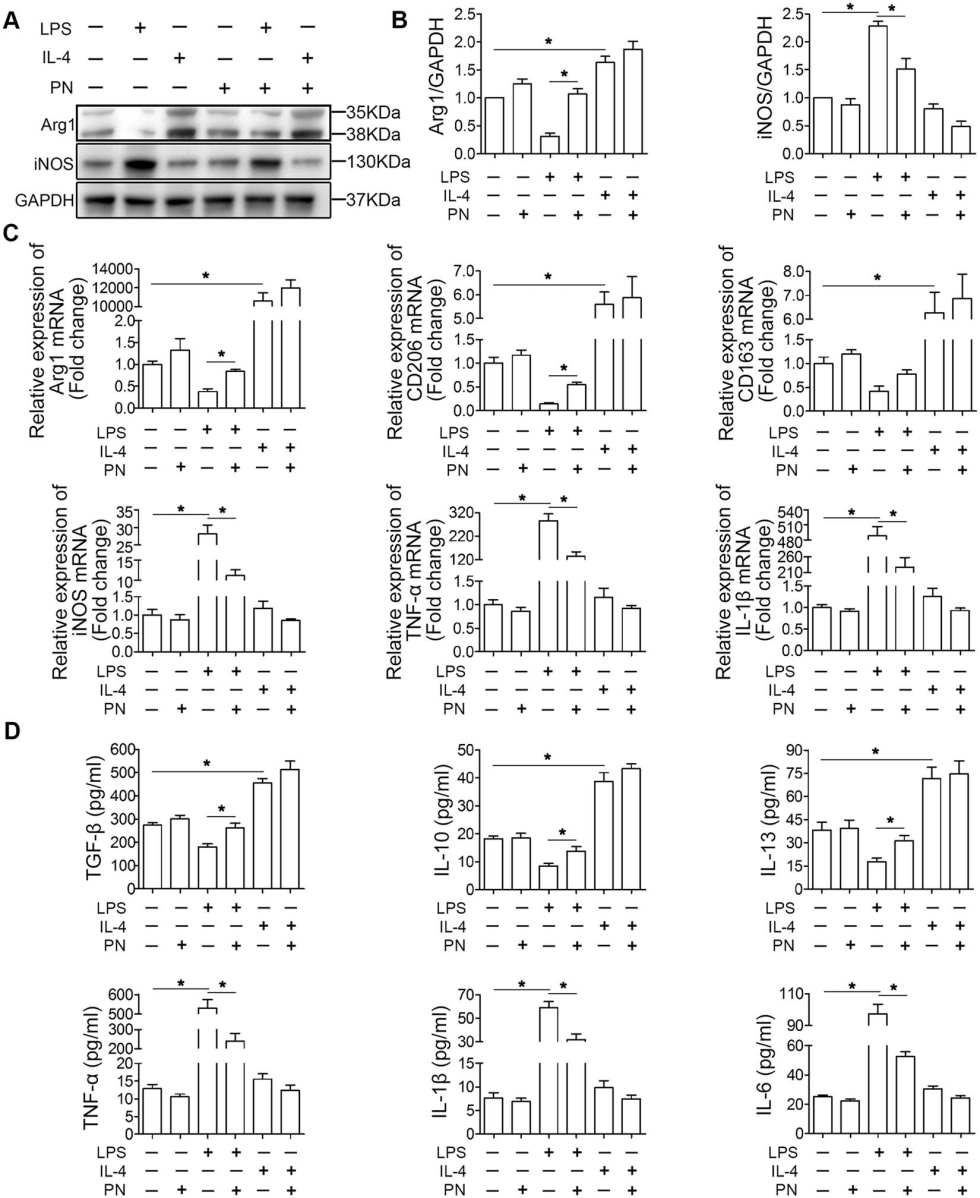
PN primarily suppressed the microglia M1 phenotype in BV2 cells in vitro. **a** Representative western blot band showed the protein levels of Arg1 and iNOS, respectively representing M2- and M1-related gene in BV2 cells under LPS or IL-4 stimulation for 24 h in the presence or absence of PN (1 μM) was detected by western blot analysis. **b** Semi-quantitative analysis of inflammation-related protein levels in **a**, which were quantified and normalized to GAPDH. (*n* = 4/group). **c** The mRNA expression levels of M2- and M1-related genes were detected by RT-qPCR (*n* = 9/group). **d** The concentration of pro-inflammatory and anti-inflammatory cytokines secreted by BV2 cells detected by enzyme-linked immunosorbent assay (ELISA). (*n* = 6/group). All data are presented as means ± SEM. *P < 0.05.

To determine whether PN exert similar therapeutic effects to those observed in vivo, LPS or IL-4 was added to culture systems for 24 h with or without the addition of PN. Firstly, according to the results of CCK8, we selected the optimal PN intervention concentration about 1 μM for in vitro study, which did not affect the primary cultured neurons and BV2 cells in vitro (Supplementary Fig. 1A, B). After 24 h of treatment of BV2 cells under different conditions, we detected the protein expression of Arg1 (M2-related gene) and iNOS (M1-related gene), by western blot analysis (Fig. 5a, b). The result showed that administration of PN reduced the expression of M1 marker and partially reversed the decrease in the expression of M2 markers in BV2 cells induced by LPS, but not affected the M1/M2 polarization under IL-4 stimulation. PCR Analysis confirmed these results via analyzing the mRNA expression of M2-related genes (Arg1, CD206, CD163) and M1-related genes (iNOS, TNF-α, and IL-1β) (Fig. 5c). Furthermore, PN promoted the secretion of anti-inflammatory cytokines (TGF-β, IL-10, and IL-13) and reduced the concentration of pro-inflammatory cytokines (TNF-α, IL-1β, and IL-6) in the culture supernatants in BV2 cells measured by ELISA (Fig. 5d), which was again confirmed the above results (Fig. 4a–c).

**Fig. 6:**
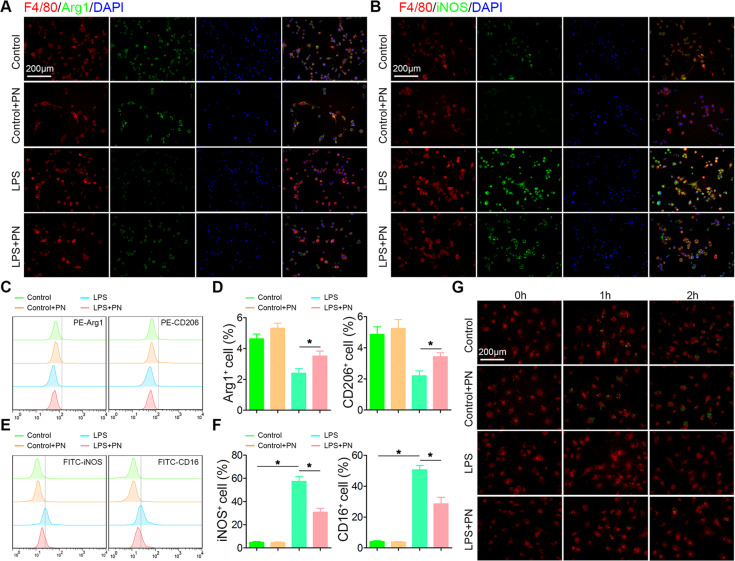
PN inhibited M1 phenotype in BV2 cells in vitro. **a**, **b** Representative images of F4/80 (red) and Arg1/iNOS(green) immunohistochemical staining of BV2 cells under LPS stimulation for 24 h in the presence or absence of PN (1 μM). (*n* = 6/group). All cell nuclei were counterstained with DAPI (blue). Scale bar, 200 μm. **c**, **e** Representative flow cytometry histograms of Arg1^+^, CD206^+^, iNOS^+^, or CD16^+^ BV2 cells under the same stimulation previously described in **a**. **d**, **f** Semi-quantitative analysis of Arg1^+^, CD206^+^, iNOS^+^, or CD16^+^ BV2 cells in **c**, **e**. (*n* = 6/group). All data are presented as means ± SEM. *P < 0.05. **g** Representative images of phagocytosis of pHrodo-Green-dye–labeled C. albicansin BV2 cells stained with F4/80 (red) followed the time course. (*n* = 6/group). Scale bar, 200 μm.

In order to further confirm the effect of PN on LPS-induced microglia polarization, we used immunofluorescence to indentify that PN reduced the expression of iNOS, and partially increased the levels of ARG1 expression in microscopic view (Fig. 6a, b). The results of flow cytometry analysis discovered that PN restored the proportion of ARG^+^ or CD206^+^ cells, and significantly decreased the proportion of iNOS^+^ or CD16^+^ positive cells under LPS stimulation for 24 h (Fig. 6c–f). To further evaluate the phagocytic ability of microglia, which is mainly thought to be one of the characteristics of M2 microglial phenotype^37^, pHrodo-Green-dye–labeled C. albicans were used to detect microglial phagocytosis in BV2 cells stimulated with LPS in the presence or absence of PN as described previously^38^ (Fig. 6g). We found that the phagocytic function in the PN exposed group was much higher than that in the control group. The phagocytosis of microglia almost disappeared after LPS induction; nevertheless, coincubated with PN and LPS, the phagocytosis of microglia was significantly raised, which was still lower than that of the control group. These combined results suggest that PN plays a robust role in balancing the polarization state of microglia M1 phenotype and M2 phenotype in vitro, which confirmed the results observed in vivo.

### PN reduced the expression of HDAC1, and suppressed NF-κB and STAT1/3 signal pathways in BV2 cells stimulated with LPS

**Fig. 7:**
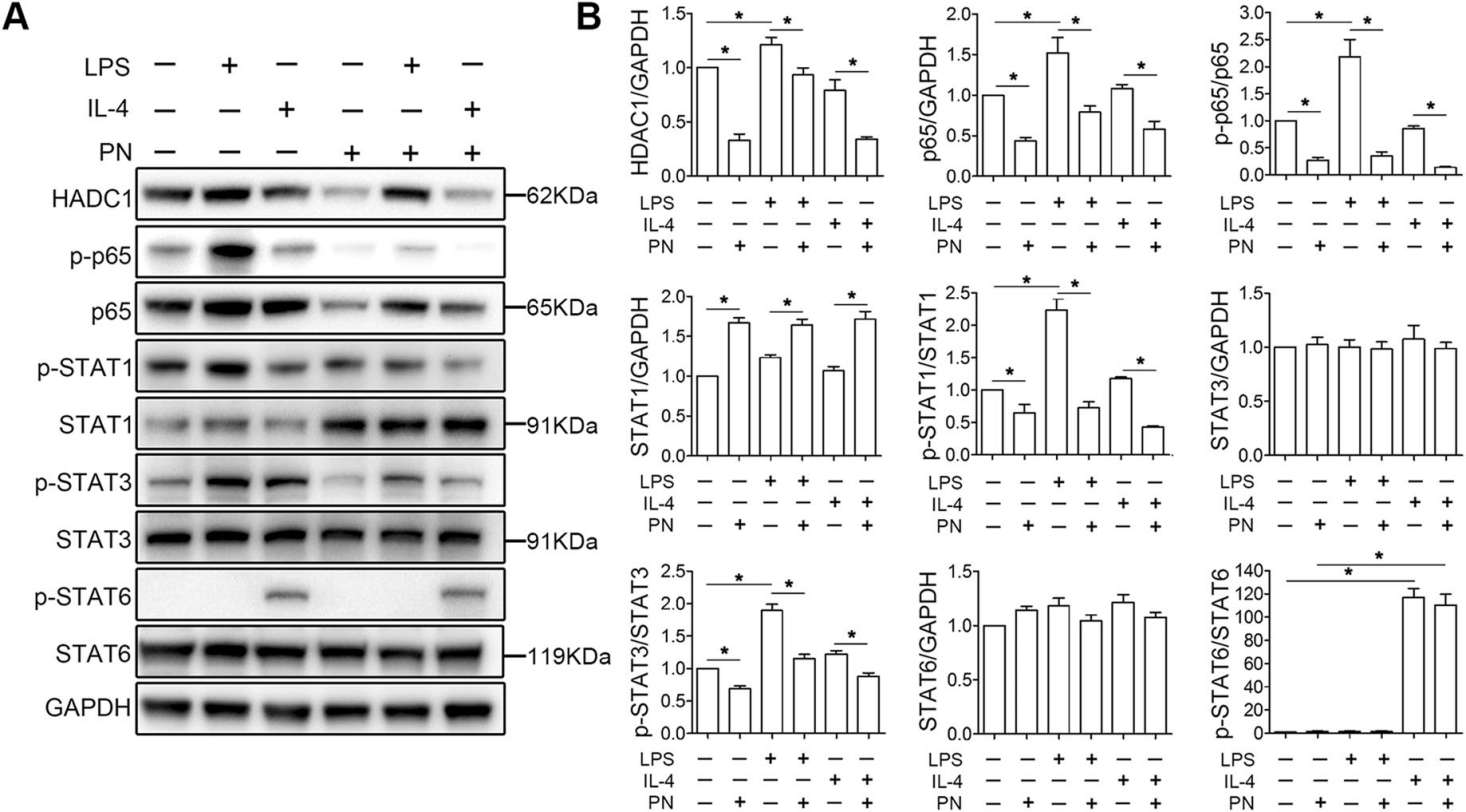
PN inhibited the expression of HDAC1, and suppressed NF-κB and STAT1/3 signal pathways. **a** Representative western blot band showed the levels of total protein (HDAC1, p65, STAT1, STAT3, and STAT6) and phosphorylated protein for the indicated molecules (p65, STAT1, STAT3, and STAT6) in BV2 cells under LPS or IL-4 stimulation for 24 h in the presence or absence of PN (1 μM). **b** Semi-quantitative analysis of the levels of total protein (HDAC1, p65, STAT1, STAT3, and STAT6) and phosphorylated protein for the indicated molecules (p65, STAT1, STAT3, and STAT6) in **a**, which were quantified and normalized to GAPDH. (*n* = 4/group). All data are presented as means ± SEM. *P < 0.05.

According to previous studies^13,18^, PN could suppress NF-κB signal pathway and specifically induce the degradation of HDAC1. We then investigated whether PN played a role in BV2 cells by western blot, and the result showed that PN downregulated the expression of HDAC1 and p65, and inhibited p65 phosphorylation under physiological conditions, LPS, or IL-4 stimulation with accompanied by a decrease in p65 protein levels (Fig. 7a, b). Interestingly, we also found a significant increase in the expression of HDAC1 and p65 after LPS stimulation compared to the control group. To our knowledge, activation of NF-κB (p65)^39,40^, STAT1^39–41^ and STAT3^42^ promotes M1 polarization, while STAT6 phosphorylation is involved in the induction of M2 polarization^40,43^. Therefore, we further explored whether PN affected the activation of STATs in BV2 cells in vitro. After PN treatment, only a increase was observed in the expression of STAT1 and no change in the expression of STAT3 and STAT6. In contrast with the control group, we observed a significant increase in the levels of p-STAT1/3 under LPS stimulation, and in the levels of p-STAT6 under IL-4 stimulation, respectively. In the presence of PN, the levels of p-STAT1/3 were significantly decreased under both LPS and IL-4 stimulation, while STAT6 phosphorylation remains unchanged. Based on these results, we speculate that PN probably regulates M1/M2 polarization of BV2 cells by inhibiting the NF-κB and STAT1/3 signaling pathways.

### PN regulated the acetylation of STATs and the binding between STAT1 and p65

**Fig. 8:**
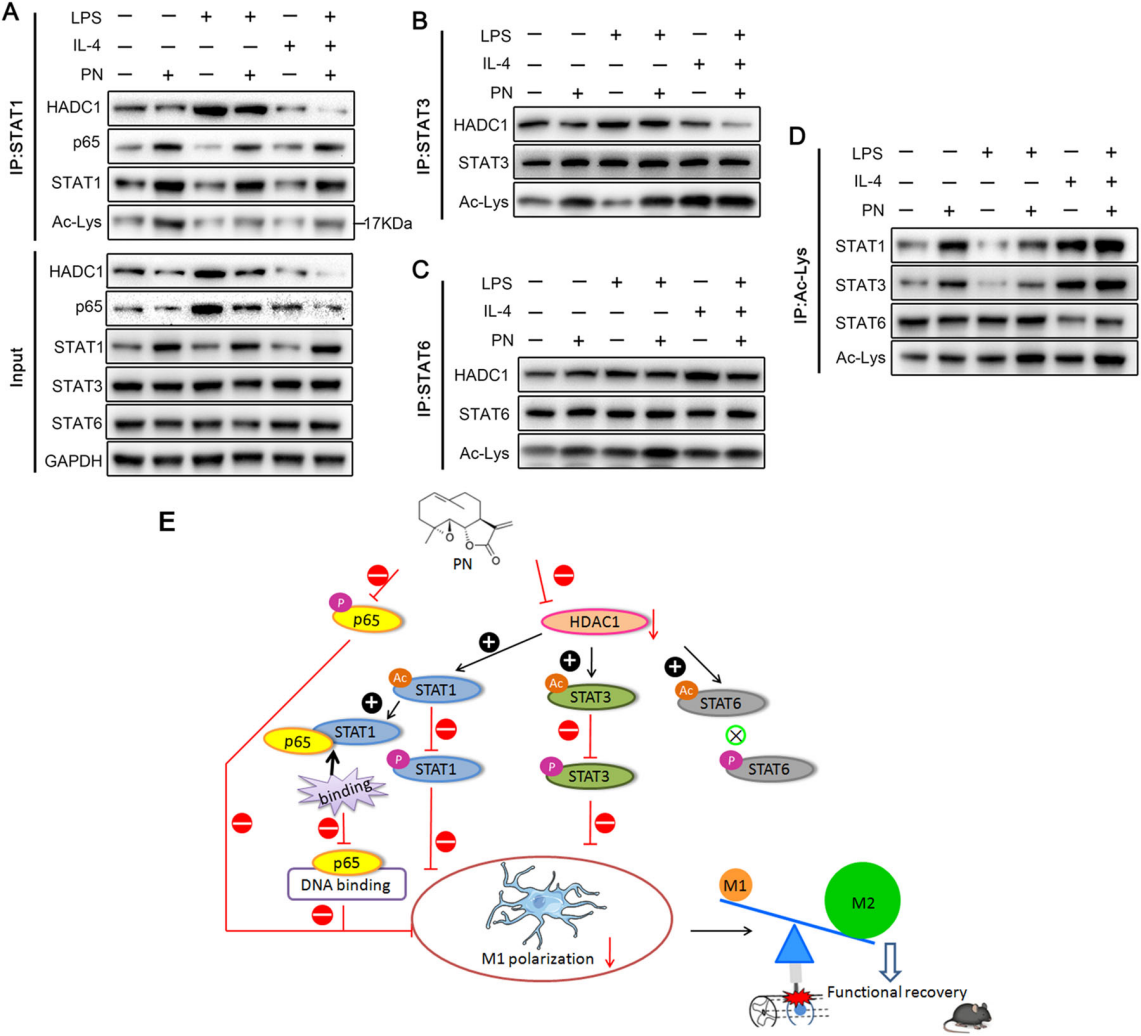
PN increased the acetylation of STATs via decreasing the binding of STATs to HDAC1, and attenuated the interaction between STAT1 and p65. **a** BV2 cells were subjected to LPS or IL-4 stimulation for 24 h in the presence or absence of PN (1 μM). Total cell lysates were coimmunoprecipitated with STAT1 and probed for HDAC1, p65, STAT1, and acetylated-lysine. **b** Total cell lysates of BV2 under the same stimulation previously described in **a** were coimmunoprecipitated with STAT3 and probed for HDAC1, STAT3, and acetylated-lysine. **c** Total cell lysates of BV2 under the same stimulation previously described in **a** were coimmunoprecipitated with STAT6 and probed for HDAC1, STAT6, and acetylated-Lysine. **d** Total cell lysates of BV2 under the same stimulation previously described in **a** were coimmunoprecipitated with acetylated-lysine and probed for STAT1, STAT3, and STAT6. (*n* = 4/group). **e** Schematic model showing the possible mechanism of PN shifting microglial/macrophages M1 polarization to M2 polarization after SCI. “+” or “−” stands for a promotion or inhibition effect, “×” stands for no influence.

Recently, increasing evidence suggests that the STATs signal pathway requires HDAC activity via modulating the acetylation of STATs^44–53^. In addition, acetylation of STAT1, is thought to be formed a complex with p65, thereby inhibiting its DNA-binding activity and downstream proinflammatory signaling cascades^54^. To evaluate the effect of PN on the acetylation of STAT1/3/6 and subsequent the interaction between STAT1 and p65, we extracted the protein lysates of BV2 cells stimulated by LPS and IL-4 for 24 h with or without PN and conducted immunoprecipitation experiments (Fig. 8a–d). The reduced combination of STAT1 and HDAC1 and the increased acetylation of STAT1 were observed in BV2 cells after PN administration whether under physiological conditions or under LPS or IL-4 stimulation, and similar effects on STAT3 and STAT6 were also showed. Meanwhile, with the increase of STAT1 acetylation, the binding of STAT1 to P65 increased as previously reported^54^. Although PN affected the acetylation of STAT6, it had no significant impact on STAT6 phosphorylation (Fig. 7) and IL-4 induced M2 polarization (Fig. 5), indicating that PN regulates M2 polarization independent of STAT6 signal. These results highlight PN switches M1 microglia to M2 polarization state mainly via suppressing NF-κB signal pathway dependent and independent of STAT1 acetylation, and decreasing phosphorylation of STAT1/3 followed by the reduced expression of HDAC1 and the subsequently increased acetylation of STAT1/3.

## Discussion

Tanacetum parthenium L. has been used as medicinal plant for over 2000 years due to its pharmacological activities, particularly the migraine prophylactic effect. Parthenolide (PN), a sesquiterpene lactone isolated from the herbal medicine feverfew is considered to be the major active constituent of the plant^55^. It has been used for the treatment of fever, migraines, arthritis, and superficial inflammation via such as antioxidative^56^ and anti-inflammatory effects^14,18^, with high safety in clinical application^57^. In addition, increasing evidences show that PN has strong anti-tumor effect^16,17^. Previous studies have showed that PN exerts neuroprotective effects by inhibiting the secretion of proinflammatory factors, resisting to oxidative stress^58^, and reducing the apoptosis pathway in the brain injury model^58,59^. PN relieves pain and promotes M2 microglia/macrophage polarization in a rat model of neuropathy^60^. To our best knowledge, there is no report of PN treatment for spinal cord injury (SCI) so far. SCI is a traumatic intractable condition with a high rate of disability and mortality. Current treatments of SCI include traditional drug therapy, surgery, cell therapy, gene therapy and tissue engineering^61–63^. However, the complicated pathophysiology of SCI includes cell death, axonal collapse and demyelination, glial scar formation, inflammation and other pathological defects. To date, although great efforts have been made, the effects of SCI treatment are still limited. Nowadays, the raised focus is on the secondary period of SCI and treatments used to suppress neuroinflammation and, as a result, create a beneficial micro-environment for neurogenesis and axonal regeneration^41^. In spinal cord, resident microglia and / or infiltrating monocytes from peripheral blood served as macrophage after SCI have long been considered two of the earliest and important participants in neuroinflammation. Although the M1 microglia/macrophage response is rapidly induced and sustained following injury, the M2 cells are transiently increased within a 1 week post-lesion period and progressively decrease thereafter^11,12^. Taking the role of the M2 phenotype with characteristics of anti-inflammation and neuroprotection into consideration, therapeutic treatments that can shift differentiated microglia from the M1 towards the M2 phenotype are encouraged in SCI^41^. The integrity of the blood–brain barrier starts to be repaired and edema begins to be resolved after 7 days^20^, and we believed that this stage was the best optimal window for PN treatment after SCI in vivo. Based on these in vitro and in vivo results, we formally found PN for the first time to promote axon regeneration and neurological function recovery by modulating the M1/M2 polarization state of microglia/macrophage known as pro-inflammatory phenotype and anti-inflammatory phenotype, respectively, which will provide a new drug option for the treatment of spinal cord injury. Experimental evidence shows that M2 microglia could reverse the detrimental effects of M1 microglia, and promote remyelination^64,65^ through upregulation of Insulin-like growth factor (IGF)-1^66^ or decreasing production of typical toxic M1 molecules such as TNF-α^67^. This indicates that an M1 to M2 switch in microglia/macrophage phenotype protects from inflammation-induced demyelination and improve neurological function after SCI^65^, which is consistent with the finding in this study.

Astrocytes, the most abundant resident cells in the CNS, play a crucial role in SCI pathology through a phenotypic change known as reactive astrogliosis. In the early stage of SCI, reactive astrocytes migrate to the lesion epicenter and seclude inflammatory cells, leading to tissue repair and functional improvement^68^. However, astrocytic scars with the accumulation of CSPG produced by astrocytes^31–33^ have been shown to be the main impediment to CNS axonal regeneration, resulting in limited functional recovery in the chronic phase of SCI^27–30^. Our research showed that PN could attenuate the hyperplasia of astrocytic scar and inhibit the expression of CSPG, which was also a possible role for axon regeneration. Recently, reactive astrocytes were further classified into A1 astrocytes and A2 astrocytes according to their functions induced by neuroinflammation and ischemia, respectively^35^. After central nervous system (CNS) injury, A1 astrocytes can secrete neurotoxins that induce rapid death of neurons and oligodendrocytes, whereas A2 astrocytes promote neuronal survival and tissue repair by secreting by several trophic factors^32,35^. As well as releasing a potent neurotoxin, A1 astrocytes were less able to promote the formation of new synapses, and caused a decrease in the excitatory function of CNS neurons^35^. Recently more and more researches indicate that A1 astrocytes may encourage the development of neurodegenerative diseases, such as Alzheimer’s disease (AD), Huntington’s disease, Parkinson’s disease, amyotrophic lateral sclerosis, and multiple sclerosis (MS), and block of A1 astrocyte conversion by microglia is neuroprotective^35,69–71^. The A2 astrocyte-related gene S100A10 is essential for cell proliferation, membrane repair, and inhibition of cell apoptosis^35^. Moreover, A2 astrocytes promote the expression of anti-inflammatory cytokine TGF-β, which participates in synaptogenesis and plays a neuroprotective role^32^. Our previous studies have shown that after SCI, inflammatory infiltration activates a new type of A1 neurotoxic reactive astrocytes, and inhibiting their activation can promote the recovery of nerve function^72,73^. Considering that type IL-1α, TNF, and C1q, known as typical proinflammatory cytokines produced from M1 phenotype of microglia^40,74^ were inducers of A1 astrocytes, and they were secreted by activated microglia under LPS stimulation^35^, which is contributed to microglia M1 polarization^40^, we speculate that the activation of M1 microglia is the key to the activation of A1 astrocytes. This study found that PN could inhibit the activation of A1 astrocytes probably via switching microglia/macrophage M1 phenotype to M2 phenotype, but other mechanisms cannot be ruled out, such as direct restraint of STAT3 activation in astrocytes, which plays a vital role in differentiation of A1 astrocytes^73^.

Previous studies^13,18^ reported that PN specifically downregulates the expression of HDAC1 and can inhibit the NF-κB signaling pathway, but the mechanism by which PN regulates microglia/macrophage M1/M2 polarization has not been clearly elucidated. Our study found that PN significantly inhibited the LPS-induced NF-κB signaling pathway in BV2 cells, and its activation is believed to promote M1 polarization^39,40^. In addition, PN inhibited the expression of HDAC1 in BV2 cells as reported^18^, whether under physiological conditions or under LPS or IL-4 induced condition. Here, it was reported that restraining activity of HDAC1 facilitates M1-to-M2 shift of microglia^75,76^. As far as we know, HDAC isoforms (HDACs) are divided into four categories: class I HDACs (1, 2, 3 and 8), class II HDACs (4–7, 9 and 10), class III sirtuins (1–7) and class IV (HDAC11). Moreover, Lcardi et al.^45^ believe that HDACs control the activation of STATs, which are the main molecules that modulate M1/M2 polarization through their own deacetylation. HDACs inhibitors, such as trichostatin A (TSA) or valproic acid (VPA) increase acetylation of STAT1 correlating with inactivation of STAT1^48^ possibly via catalyzing dephosphorylation of STAT1, known as a phosphorylation-acetylation switch mechanism^49^. It has been suggested that acetylated STAT1 interacts with NF-κB (p65) and consequently inhibits NF-κB signal pathway^54^. In this study, it was observed that increased acetylation of STAT1 was accompanied by its decreased phosphorylation and improved the association with p65, via reducing the binding of HDAC1 to STAT1 after PN administration, which suggests that PN plays an important role in LPS-induced STAT1 activation and indirect regulation of NF-κB signal by downregulating HDAC1 expression. Treatment of diffuse large B-cell lymphoma (DLBCL) with the HDAC inhibitor panobinostat (LBH589) results in STAT3 hyperacetylation, dephosphorylation and increased nuclear export, inhibiting the transcription of STAT3-responsive anti-apoptotic genes and the survival of DLBCL cells^44^. Consistently, administration of Trichostatin A (TSA) as inhibition of HDACs is also shown to increase STAT3 acetylation while suppressing phosphorylation on Tyr705 and cell proliferation^50^. And, we found that the effect of PN on STAT3 activation similar to previous reports above^44,50^ was consistent with its regulation on STAT1. Yang et al.^53^ consider that TSA also leads to a increase in the acetylation of STAT6, and inhibits the activation of STAT6 signaling pathway during mouse dendritic cell differentiation. Unlike STAT1/3, the biological consequence of STAT6 acetylation and the relationship between its acetylation and phosphorylation are not known at present. Although PN could increase the acetylation of STAT6, it did not affect the phosphorylation of STAT6, which may be related to the activation of its site-specific lysine acetylation by PN, considering that different acetylation sites phosphorylate STAT1/3 differently^52^. Thus, the acetylation sites in STAT6 and corresponding biological functions need to explore further. In vitro results suggested that PN could significantly reduced M1 polarization in BV2 cells and partially rescued the decrease in the expression of M2 phenotype markers of microglia/macrophage induced by LPS, but no significant effect on M2 polarization stimulated with IL-4 was observed. Combined with the above results, we speculate that PN facilitates shift from M1 to M2 polarization of microglia/macrophage during SCI by regulating NF-κB signal directly or indirectly and the activation of STAT1/3.

In summary, our study showed that PN could shift microglia/macrophages from M1 to M2 phenotype, which can inhibit the activation of NF-κB and STAT1/3 signaling pathway. The present study provides the first evidence for the critical role of the PN in promoting axonal regeneration, increasing myelin reconstitution, reducing CSPG formation, inhibiting scar hyperplasia, suppressing the transformation of A1 astrocytes and consequently, improving functional behavioral recovery after acute SCI by regulating microglia/macrophages polarization.

## Methods and materials

### Reagents and antibodies

The Parthenolide (PN) was purchased from MCE (Monmouth Junction, NJ, USA). The microglial activator lipopolysaccharide (LPS) was purchased from Sigma-Aldrich (St. Louis, MO, USA). Recombinant mouse IL-4 was obtained from Abcam (Cambridge, UK). Primary antibodies used in this study included rabbit anti-INOS (Abcam), rabbit anti-Arginase (Santa Cruz, Dallas, Texas, USA), mouse anti-F4/80 (Abcam), mouse anti-Neurofilament (Abcam), mouse anti-chondroitin sulfate (Abcam), rabbit anti-GFAP (Abcam), rabbit anti-MBP (Cell Signaling Technology, Danvers, MA, USA), rabbit anti-NF-κB p65 (Cell Signaling Technology), rabbit anti-p-p65 (Cell Signaling Technology), rabbit anti-C3 (Abcam), rabbit anti-GAP43 (Abcam), mouse anti-STAT1 (Santa Cruz), mouse anti-p-STAT1 (Santa Cruz), mouse anti-STAT3 (Cell Signaling Technology), mouse anti-p-STAT3 (Cell Signaling Technology), mouse anti-STAT6 (Santa Cruz), mouse anti-p-STAT6 (Santa Cruz), rabbit anti-HDAC1 (Santa Cruz), and rabbit anti-acetyl Lysine (Santa Cruz). Alexa Fluor 488 and 594 conjugated to goat anti-mouse IgG (H+L) or goat anti-rabbit IgG for immunofluorescence staining were obtained from Jackson ImmunoResearch (West Grove, PA, USA). Horseradish peroxidase (HRP)-conjugated goat anti-rabbit IgG (H+L) and HRP-conjugated goat anti-mouse IgG (H+L) for immunoblotting were purchased from Invitrogen (Carlsbad, CA, USA). Protein A/G Magnetic Beads for co-immunoprecipitation (co-IP) were obtained from Pierce Biotechnology (Rockford, IL, USA). The DeadEnd™ Fluorometric TUNEL System was purchased from Promega (Madison, WI, USA). ELISA kits for TNF-α, IL-1β, IL-6, IL-13, IL-10 and TGF-β were purchased from Abcam. The antibodies used for flow cytometry were: PE-Cyanine7-labeled anti-mouse Arg1 mAb (eBiosciences, San Diego, CA), PE-labeled anti-mouse CD206 mAb (eBiosciences), FITC-labeled anti-mouse CD16 mAb (eBiosciences), FITC-labeled anti-mouse INOS mAb (BD Biosciences, San Jose, CA, USA).

### Preparation of the contusive SCI mouse model

All animal procedures were conducted according to the Guidelines for the Care and Use of Laboratory Animals (National Institutes of Health) and approved by the Animal Care and Use Committee of Nanjing Medical University. Briefly, 16 male C57BL/6J mice (18–35 g; 8 weeks old) were first deeply anesthetized by intraperitoneal injection of pentobarbital sodium (50 mg/kg of body weight). The hair on the back of the mice was shaved off and 75% alcohol was used to disinfect the skin. The vertebral column was carefully exposed, followed by excision of the T9 lamina. The spine was stabilized by clamping the T7 and T11 spinous processes. After the dorsal surface of the cord was fully exposed, a rod (1.3 mm in diameter; RWD Life Science Corp., C4p01-001, China) was used to compress the spinal cord with a force of 50 kdynes and a dwell time of 10 s. After the moderate SCI model was created, the muscles and the skin were quickly sutured. The bladders of mice were manually and gently massaged three times a day to avoid retention of urine until the reflexive control of micturition was restored. Mice fulfilling these SCI models were randomly divided into two groups, a SCI-only group and a treatment group (*n* = 8/group). The SCI-only group served as the control (*n* = 8/group). PN was intraperitoneally injected at a dose of 2 mg/kg daily referred to previous reports^77,78^ for 7 days in the treatment group for subsequent analysis.

### Tissue processing

Mice were sacrificed with an overdose of pentobarbital sodium (80 mg/kg, intraperitoneally) on day 3, 7, and 14 post-injury. The hearts of mice were immediately perfused by 10 ml ice-cold physiological saline followed by ice-cold physiological saline plus paraformaldehyde (4%, w/v). The incision of SCI was opened and segments of spinal cord containing the lesion site were extracted. Spinal cord samples were then fixed in paraformaldehyde (4%, w/v) at 4 °C for 24 h, dehydrated in a sucrose-PBS gradient (20 and 30%, w/v). Finally, the samples were embedded in OCT, and cut into serial longitudinal sections of about 10 µm. All sections were stored at −80 °C until processed for immunostaining.

### Assessment of locomotor capacity

The motor function after SCI was quantified at 1, 3, 7, 14, 21, and 28 day after surgery using the Basso Mouse Scale (BMS) for locomotion. Scoring ranged from 0 for complete paraplegia to 9 for normal function.

### Footprint analysis

Gait and motor coordination were assessed at 3, 14 and 28 day after surgery. The front and rear paws were painted with different colors of dye. The mouse was then placed on a piece of blotting paper surrounded by a cage to encourage it to walk in a straight line. The footprint pattern was digitized and a representative picture was used to assess coordination.

### BV2 cell culture and treatment

The BV2 microglial cell line was purchased from the Cell Bank of the Chinese Academy of Science (Shanghai, China). Briefly, the BV2 cells were cultured in Dulbecco’s modified Eagle’s medium (DMEM, Thermo Fisher Scientific, Waltham, MA, USA) supplied with 10% fetal bovine serum (FBS, Thermo Fisher Scientific), 100 units/ml penicillin, and 100 μg/ml streptomycin (Thermo Fisher Scientific) at 37 °C with 5% CO_2_. The complete DMEM medium was changed once a day and cells were passed every 3 days. When the BV2 cells grew to a confluence of about 80%, the medium was replaced with serum-free DMEM, and incubated with LPS (100 ng/ml)/IL-4 (20 ng/ml) with or without PN (1 μM) according to CCK8 result for 24 h. Then the BV2 cells were used for further experiments.

### Cell viability

Primary neurons were prepared from WT C57/BL6 embryonic day 15 mice according to an established protocol^24^. Viability of primary neurons and BV2 cells as evaluated with a Cell Counting Kit-8 (CCK-8) assay (Dojindo, Kumamoto, Japan) to examine the effect of PN on viability assessment. After 0, 6, 24, and 48 h of incubation with different PN concentration, Wells were rinsed 3 times with PBS, and CCK8 solution (10 ml; 1:10 dilution) in fresh culture medium was added to wells and incubated for 2 h at 37 °C. The optical absorbance of each sample at 450 nm was measured using a microplate reader (ELx800; Bio-Tek, Winooski, VT, USA). Cell viability was expressed as the dehydrogenase activity in cells relative to that in the PBS control group.

### Western blot analysis

Proteins were extracted from cells and treated with RIPA lysis and extraction buffer (KeyGen Biotechnology, Shanghai, China). Protein concentration was determined using the BCA method. Equal amounts of protein were separated by SDS-PAGE, transferred to PVDF membranes (EMD Millipore Corp., Burlington, MA), and incubated overnight at 4 °C with primary antibodies followed by blocking with 5% bovine serum albumin (BSA, KeyGen Biotechnology). Membranes were then incubated for 120 min at room temperature with the secondary antibody. Reacting bands were visualized using ECL reagent (Thermo Fisher Scientific), and the density of protein bands was semiquantified using Image J (National Institutes of Health, Bethesda, MD, USA).

### Co-immunoprecipitation (CO-IP)

After treatment, the cell samples were lysed in 1 ml ice-cold buffer A (20 mM TrisHCl, pH 7.4, 150 mM NaCl, 1% Triton X-100, 0.5% sodium deoxycholate, 12 mM glycerophosphate, 10 mM sodium fluoride, 5 mM EGTA, 2 mM sodium vanadate, 1 mM PMSF, 2 mg/ml aprotinin, and 2 mg/ml leupeptin) for 30 min at 4 °C. Lysates were then centrifuged at 14,000 rpm for 15 min to collect the supernatants. The supernatants were immunoprecipitated with the indicated antibodies overnight at 4 °C using protein A/G Magnetic Beads. The beads were rinsed three times with buffer A and the eluent proteins were subjected to 12% SDS-PAGE for separation and transferred to polyvinylidene difluoride (PVDF) membranes for immunoblotting as described in western blot analysis.

### Immunofluorescence staining

Spinal cord tissues or cultured BV2 cells were fixed with precooled 4% paraformaldehyde for 20 min at 4 °C, then permeabilized with 0.2% Triton X-100 for 20 min, and blocked with 10% normal goat serum for 0.5 h. Fixed tissues and cells were then incubated at 4 °C overnight with primary antibodies, followed by Alexa Fluor 488– and Alexa Flour 594–conjugated goat secondary antibody (Jackson ImmunoResearch) for 1 h at room temperature. After triple washing by PBS, nuclei were stained with DAPI (Thermo Fisher Scientific) and fluorescent images were acquired using an epifluorescence microscope (AxioVertA1 and ImagerA2). The lesion volume was obtained by the sum of total lesion according to GFAP^+^ lesion cavity area multiplied by distance (about 200 μm) between the sections.

### Phagocytosis assay

PHrodo-Green-dye–labeled C. albicans (Invitrogen, Carlsbad, CA, USA) were used to access the phagocytosis ability of BV2 cells at the presence or absence of PN. Briefly, 2 × 10^6^ BV2 cells were seeded in each well of 24-well plates. After the cells adhere to the wall, the medium was replaced with serum-free DMEM, and incubated with LPS (100 ng/ml) with or without PN (1 μM) for 24 h. Then the supernatant was replaced by a suspension of pHrodo-labeled C. albicans at a 1:5 ratio (progenitor: yeasts) in 1 ml serum-free DMEM for the indicated durations. At indicated time points, the C. albicans suspension was removed and the cells were rinsed with PBS for 3 times. Then the cells were fixed, permeabilized and blocked, and incubated with primary antibody of F4/80 followed by Alexa Flour 594–conjugated goat secondary antibody (Jackson). The mean green fluorescence intensity of engulfed bacteria was estimated by an epifluorescence microscope (AxioVertA1 and ImagerA2).

### Flow cytometry (FCM) analysis

After incubation with LPS (100 ng/ml)/IL-4 (20 ng/ml) with or without PN (1 μM) for 24 h, the rate of BV2 cells transition to different polarized phenotypes was measured by flow cytometry. Briefly, the culture medium was discarded followed by three washes with PBS. BV2 cells were digested using 0.25% trypsin to prepare a single-cell suspension. The suspension was then incubated with indicated antibodies at 4 °C under darkness for 30 min. Cells were harvested by centrifugation at 1500 rpm for 5 min, and washed twice with PBS. Finally, the suspension was analyzed by flow cytometry (FACSCalibur, BD Biosciences, Franklin Lakes, NJ, USA).

### Real-time reverse transcription polymerase chain reaction (RT-qPCR)

Total RNA was extracted from BV2 cells after different treatments using Trizol Reagent (Invitrogen) according to the manufacturer’s instructions. The concentrations of total RNA were measured by a Biometra Optical Thermocycler (Analytik Jena, Goettingen, Germany). RNA (500 ng) was reverse transcribed into cDNA using the High Capacity cDNA Reverse Transcription Kit (Thermo Fisher Scientific) according to the manufacturer’s instruction. The primer sequences used for qPCR amplification were:

TNF-α: 5′-CCCTCACACTCAGATCATCTTCT-3′ (forward)

5′-GCTACGACGTGGGCTACAG-3′ (reverse).

IL-1β: 5′-GCAACTGTTCCTGAACTCAACT-3′ (forward)

5′-ATCTTTTGGGGTCCGTCAACT-3′ (reverse).

iNOS: 5′-CTCCTTCAAAGAGGCAAAAATA-3′ (forward)

5′-CACTTCCTCCAGGATGTTGT-3′ (reverse).

Arg-1: 5′-CCTGGAACTGAAAGGAAAG-3′ (forward)

5′-TTGGCAGATATGCAGGGAGT-3′ (reverse).

CD206: 5′-CTGCAGATGGGTGGGTTATT-3′ (forward)

5′-GGCATTGATGCTGCTGTTATG-3′ (reverse).

CD163: 5′-GCC ATA ACT GCA GGC ACA AA-3′ (forward)

5′-GTT GGT CAG CCT CAG AGA CA-3′ (reverse).

GAPDH: 5′-TGTGATGGGTGTGAACCACG-3′ (forward)

5′-CAGTGAGCTTCCCGTTCACC-3′ (reverse).

Quantitative real-time PCR was performed using SYBR qRCR premix (Takara, Kyoto, Japan) according to the manufacturer’s instructions. Cycling conditions were an initial denaturation at 95 °C for 30 s, followed by 40 cycles at 95 °C for 5 s, 60 °C for 30 s, and 72 °C for 10 min. Target gene expression was normalized to GAPDH expression (internal control) using the ∆∆CT method.

### ELISA

The concentrations of TNF-α, IL-1β, IL-6, IL-13, IL-10, TGF-β were measured in BV2 culture media after indicated treatments using ELISA kits according to the manufacturers’ protocols. Briefly, the culture medium of BV2 cells was collected and centrifuged at 2000 *g* for 10 min to remove debris. 50 µl of all samples or standard was added to appropriate wells followed by 50 µl of the Antibody Cocktail to each well. The samples were incubated for 1 h at room temperature on a plate shaker at 400 rpm. Then all wells were washed three times with 1×Wash Buffer PT followed by 100 µl/well of TMB substrate and incubated under darkness for 10 min. Finally, STOP Solution was added and absorbance was read at 450 nm wavelengths using a microplate reader (ELx800, Bio-Tek, USA).

### Nissl staining of spinal cord slices

The cytosolic Nissl substance in spinal cord sections was stained with cresol violet on the 28th day after surgery. In brief, the slices were washed with distilled water and stained in a cresol violet solution for 10 min. After rinsing with distilled water, the slices were differentiated with 95% ethanol, rinsed with xylene, and fixed with neutral balsam. Regions of traumatic injury were identified by severe tissue destruction or staining loss. Six Nissl-stained sections by distance (about 200 μm) between the sections were selected to estimate the average number of residual anterior horn motor neurons and the proportional lesion size.

### Statistical analyses

Results are expressed as mean ± SEM of at least three independent experiments. Means were compared by one-way ANOVA followed by Bonferroni’s post hoc tests for multiple comparisons (SPSS 20; SPSS, Chicago, IL, USA). A *p* < 0.05 (two tailed) was regarded as significant for all tests.

## Supplementary information

summary of supplemental information

supplemental f1

Supplemental Materials

uncropped gels

## Data Availability

The data that support the findings of this study are available from the corresponding author upon reasonable request.
