# Supplementary material for: Parthenolide promotes the repair of spinal cord injury by modulating M1/M2 polarization via the NF-κB and STAT 1/3 signaling pathway
Source: Cell Death Discov. 2020 Oct 6;6:97. doi: 10.1038/s41420-020-00333-8 (PMC7538575; doi:10.1038/s41420-020-00333-8)
Supplement: Supplementary file 1 — summary of supplemental information [file 41420_2020_333_MOESM1_ESM.docx]

**summary of supplementary information**

1. supplemental f1：CCK-8 assay of effects of PN on the viability and activation of primary neurons and BV2 cells
2. Supplemental Materials：the figure legend of supplemental f1.
3. uncropped gels.ZIP: uncropped gels indicating how the figure was prepared should be included.
