# Supplementary figures and images for: Parthenolide promotes the repair of spinal cord injury by modulating M1/M2 polarization via the NF-κB and STAT 1/3 signaling pathway
Source: Cell Death Discov. 2020 Oct 6;6:97. doi: 10.1038/s41420-020-00333-8 (PMC7538575; doi:10.1038/s41420-020-00333-8)

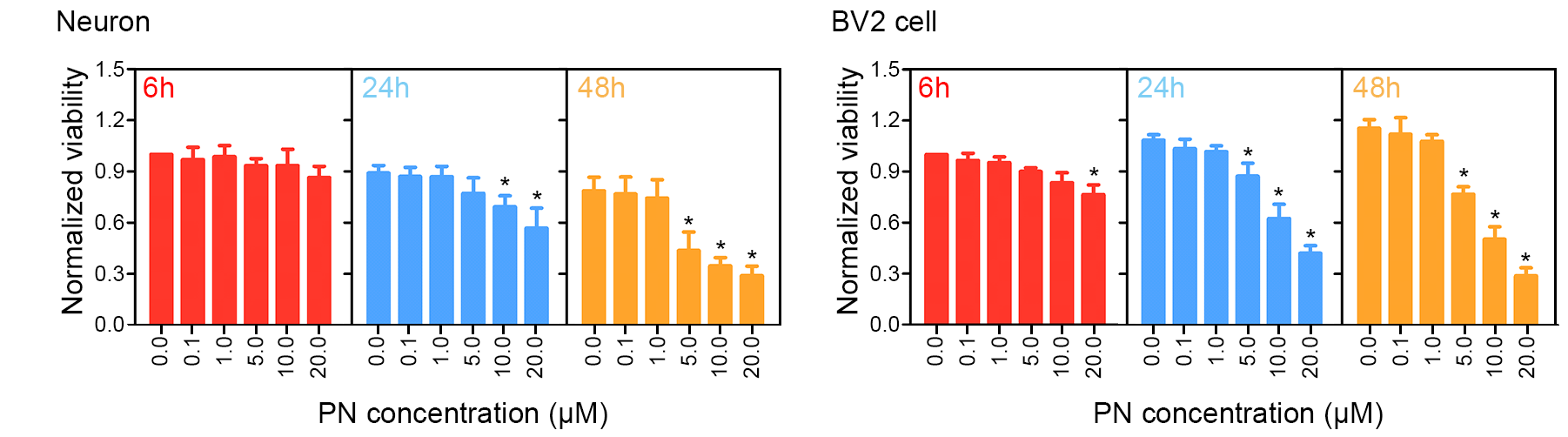

Supplement: Supplementary file 2 — supplemental f1 [file 41420_2020_333_MOESM2_ESM.tif]
