# Supplementary material for: Parthenolide promotes the repair of spinal cord injury by modulating M1/M2 polarization via the NF-κB and STAT 1/3 signaling pathway
Source: Cell Death Discov. 2020 Oct 6;6:97. doi: 10.1038/s41420-020-00333-8 (PMC7538575; doi:10.1038/s41420-020-00333-8)
Supplement: Supplementary file 3 — Supplemental Materials [file 41420_2020_333_MOESM3_ESM.doc]

Supplemental Figure1. Effects of PN on the viability and activation of primary neurons and BV2 cells. Primary neurons (A) and BV2 cells (B) were incubated with different concentrations of PN for 6h, 24h, and 48h. Cell viability was determined using CCK-8 assay. All data are presented as means ± SEM (n = 5 / group). *P＜0.05.
